# Perception of the Food Environment and Food Security Levels of Residents of the City of Rio de Janeiro

**DOI:** 10.3390/ijerph22040642

**Published:** 2025-04-18

**Authors:** Paulo César Pereira de Castro Junior, Yoko Ametista Carvalho Suéte Matos, Roberta Teixeira de Oliveira, Rosana Salles-Costa, Aline Alves Ferreira

**Affiliations:** 1 Applied Social Nutrition Department, Josué de Castro Nutrition Institute, Federal University of Rio de Janeiro, Rio de Janeiro 21941-902, Brazil; rosana@nutricao.ufrj.br (R.S.-C.); alineaf@nutricao.ufrj.br (A.A.F.); 2 Josué de Castro Nutrition Institute, Federal University of Rio de Janeiro, Rio de Janeiro 21941-902, Brazil; yokoametista@ufrj.br (Y.A.C.S.M.); oliveira.robertta2@ufrj.br (R.T.d.O.)

**Keywords:** food insecurity, food environment, Brazil, inequalities

## Abstract

The way individuals perceive and interact with the food environment can contribute to a higher prevalence of food insecurity (FI). Objective: To evaluate the perception of the food environment and its association with FI in households in the city of Rio de Janeiro, Brazil. Methods: Cross-sectional study. The survey was conducted with a sample of 2000 households, a representative stratified sample, with a margin of error of 4.9 percentage points and a 95% confidence interval (CI95%) in the city of Rio de Janeiro. The studies were evaluated using the Brazilian Food Insecurity Scale (EBIA). Perceptions of the food environment were measured by assessing the perceived availability, price, and quality of fruits and vegetables (FVs) and ultra-processed foods (UPFs) sold in the neighborhood. To analyze the association between stage variations and the perceived food environment, we conducted multinomial logistic regression, considering a 95%CI. Results: Household heads in Rio de Janeiro perceive that both FVs and UPFs are available in their neighborhoods. However, UPFs are perceived as cheaper and more diverse than FVs, regardless of the level of food safety. In the association analysis, a greater relative risk ratio was found for heads of households who perceive an unfavorable scenario in the food environment for FVs, in terms of availability (RRR = 5.6; 95%IC: 3.0–10.4), quality (RRR = 4.5; 95%IC: 2.6–7.9), and price (RRR = 2.5; 95%IC: 1.7–3.6), to experience a situation of moderate/severe FI. Conclusions: The way individuals interact with and perceive their territories can reflect on access to adequate and healthy food, especially in households in a situation of FI.

## 1. Introduction

The food environment refers to the physical, economic, political, and socio-cultural context in which consumers interact with the food system to acquire, prepare, and consume food [1,2]. It encompasses the physical spaces where food is obtained and the resources and infrastructure that facilitate access to these locations, and can be influenced by individual factors such as income and education, as well as by public and commercial policies that shape food choices [2,3].

Globally, food environments have been undergoing profound transformations driven by processes such as urbanization, the globalization of food systems, and changes in patterns of food production, distribution, and consumption. These transformations occur unevenly across countries and within territories, creating contexts in which ultra-processed foods are predominantly available, while physical and economic barriers to accessing fresh or minimally processed foods persist—especially among populations in situations of vulnerability [1,4]. This dynamic poses obstacles to the realization of the human right to adequate food and undermines the achievement of the Sustainable Development Goals (SDGs), particularly those related to Zero Hunger (SDG 2) and the reduction in social inequalities (SDG 10) [5].

In this context, the way individuals perceive the food environment in which they are embedded becomes particularly relevant. This perception is subjective, varying according to personal experience and the social and cultural conditions in which an individual exists [6,7]. This process involves how factors such as the accessibility, availability, quality, variety, price, and convenience of food in one’s surroundings are interpreted [8,9,10]. Elements such as proximity to food retail stores, food costs, and the quality of service at points of sale directly impact perception [11]. The perception of the availability and quality of foods considered to be healthy, such as fruits and vegetables (FVs), may be positively associated with the increased consumption of these foods, as indicated by some authors [7,12,13].

Adequate and healthy nutrition is a right guaranteed by law in Brazil [14], such as continuous and systematic access to food in a sufficient quantity and quality while respecting diverse sociocultural characteristics [15]. Any difficulty families face in securing this right constitutes a scenario of food insecurity (FI) [16,17,18]. Therefore, there is a direct relationship between the socioeconomic and demographic conditions of households, FI, Food and Nutrition Security, and the food environment.

A condition of FI is that it can be exacerbated by unfavorable conditions in the food environment [19,20,21]. Factors such as a lack of financial resources, a scarcity of healthy options nearby, difficulties in accessing retail food in the neighborhood, or the absence of effective public policies that ensure access to adequate food contribute to the occurrence of food insecurity.

In the municipality of Rio de Janeiro, one of the largest metropolises in Brazil and Latin America, data from the First Survey on Food Insecurity in the City of Rio de Janeiro showed that in 2024, 32.9% of households faced some level of FI. Among them, 16.0% experienced moderate FI (quantitative restriction of food), and 7.9% were in a situation of severe FI (hunger), distributed unevenly across the city [22].

There is evidence of a relationship between FI and territorial inequality in Rio de Janeiro, which is also linked to disparities in the food environment and perceptions of it. Studies indicate that the perceived ease of purchasing and the variety of ultra-processed foods (UPFs) tend to be higher in households experiencing FI compared to those of fruits and vegetables (FVs) [23]. Thus, the present study aimed to identify perceptions of the food environment and its association with levels of FI in households in the municipality of Rio de Janeiro, one of the largest metropolises in Latin America.

## 2. Materials and Methods

### 2.1. Study Design and Location

This is a cross-sectional study, which was conducted for the first time in one of Brazil’s main metropolises using data from the study titled “I Survey on Food Insecurity in the City of Rio de Janeiro”. The study was carried out between November 2023 and January 2024 and aimed to assess the levels of FI in the municipality of Rio de Janeiro, Brazil [22].

Rio de Janeiro is a municipality with approximately 6,211,233 inhabitants and a population density of 5174.6 inhabitants per km^2^, according to data from the 2022 census [24]. Territorially, it is divided into 5 planning areas, encompassing its 165 neighborhoods. The average per capita income is USD 880.81, equivalent to a 3.6 Brazilian minimum wage [24]. However, it is one of the most economically unequal cities in Brazil.

### 2.2. Data Source, Population, and Sampling

This is a population-based survey that assessed a sample of 2000 households, representative of the municipality of Rio de Janeiro, distributed across census sectors considering the neighborhoods of the five planning areas (PAs) into which the municipality is divided. The sample size considered a prevalence of 15.9% for severe FI in the state of Rio de Janeiro [25]. The design adopted a 95% confidence level and a maximum margin of error of 4.9 percentage points within each PA. For the overall sample, the maximum margin of error was 2.2 percentage points. The sampling design and procedures are further described in the First Survey on Food Insecurity in the City of Rio de Janeiro [22]. In this study, a sample of 1819 households had complete data for the principal outcomes and variables of study (9.0% of missing values; n = 181). 

The interviews were conducted using a mobile device application by properly trained professionals—hired by a company specializing in data collection—and were preferably administered to the head of the household (over 18 years old and capable of answering questions about the household’s socioeconomic and dietary profile). The questionnaire consisted of the following modules: (1) household and family identification; (2) family profile; (3) income and employment; (4) Brazilian Food Insecurity Scale (EBIA); (5) social support and food-related issues; (6) information about the place of residence (neighborhood perception); (7) information about food acquisition locations; and (8) nutritional status. For this study, modules 1, 2, 3, 4, and 6 were considered in the analysis.

### 2.3. Study Variables

#### 2.3.1. Household Food Insecurity

The outcome variable, FI, was measured using the Brazilian Food Insecurity Scale (EBIA), a validated scale for measuring FI in the Brazilian context [26]. The EBIA was employed to classify households into the following mutually exclusive categories of food security and FI. Food security refers to a condition in which a household has permanent access to food of adequate quality and quantity. Mild FI is characterized by concern or uncertainty regarding future access to food. Moderate FI occurs when there is a quantitative reduction in food intake among adults and/or a disruption in eating patterns due to a lack of food. Severe FI involves a quantitative reduction in food intake among both adults and individuals under 18 years of age, that is, a disruption in eating patterns due to food scarcity affecting all household members; in this context, hunger becomes an experienced reality within the household.

The EBIA comprises fourteen dichotomous questions (‘yes’ or ‘no’), including eight items applicable only to households with adults (aged 19 years or older) and six items relevant to households with children and/or adolescents. Food security/FI levels are determined based on the total number of affirmative responses. Accordingly, the cut-off points for households with individuals under 18 years of age are as follows: 0—food security; 1–5—mild FI; 6–9—moderate FI; and 10–14—severe FI. For households without individuals under 18 years of age, the cut-off points are as follows: 0—food security; 1–3—mild FI; 4–5—moderate FI; and 6–8—severe FI [26].

The EBIA is the official Brazilian government tool for assessing household FI in national representative surveys [27,28]. Its reliability and validity for assessing household FI in Brazil are well documented in several international studies [29,30,31]. An important aspect of the EBIA is that it has helped harmonize the understanding of the construct of FI among key stakeholders, ranging from civil society organizations to government leaders [32].

In this study, household FI was categorized into food security and two levels of FI (mild FI (MFI), and moderate or severe FI (MSFI)), following the methodology of previous studies [23,33].

#### 2.3.2. Dimensions of the Perception of the Food Environment in the Neighborhood

To assess perceptions of the food environment, a questionnaire from a national study [34] was used, involving statements about the perception of access to fruits and vegetables (FVs) and ultra-processed foods (UPFs), composed of eight questions: “It is easy to buy fruits and vegetables in your neighborhood”; “Fruits and vegetables are of good quality in your neighborhood”; “There is a wide variety of fruits and vegetables in your neighborhood”; “Fruits and vegetables are cheap in your neighborhood”; “It is easy to buy soft drinks, cookies, packaged snacks, sweets, and other treats in your neighborhood”; “There is a wide variety of soft drinks, cookies, packaged snacks, sweets and other treats in your neighborhood”; “Soft drinks; cookies; packaged snacks; candy and other treats are cheap in my neighborhood.”; and “Fast food meals are cheap in my neighborhood.”.

The head of the household could respond by indicating their level of agreement with each statement on a five-point Likert scale, ranging from “strongly disagree” to “strongly agree”. For this article, the variables “strongly agree” and “partially agree” were grouped, as well as “strongly disagree” and “partially disagree”, as proposed by Justiano et al. (2024) [23].

The socioeconomic characteristics of the heads of the household analyzed in this study included the following: sex (female/male), age (18–35; 36–45; 46–60; 60 or older), education level (elementary; high school; university), and race/skin color, composed of self-declared black, mixed-race, and white individuals. Indigenous and Asian categories were excluded due to a lack of data or low representativity. Individuals who self-identified as black or mixed-race were grouped and analyzed together under a single race/skin color category [35,36,37]. Regarding the household, the per capita family income was classified into the following categories: up to ½ minimum wage, ½ to 1 minimum wage, more than 1 up to 2 minimum wages, more than 2 minimum wages, and the PAs (PA1—Central Zone; PA2—South Zone and Tijuca; PA3—North Zone; PA4—Barra da Tijuca and Jacarepaguá; and PA5—West Zone).

### 2.4. Statistical Analysis and Ethical Permissions

Initially, descriptive analyses were conducted with percentage estimates of the main variables in the study (per capita income and sociodemographic variables), according to the study’s outcome variable (food security/FI), using a statistical significance test to assess the relationship and differences between them (Chi-square test).

The description of food environment perceptions was evaluated for the entire sample and stratified by the households’ FI levels (food security, mild FI, and moderate or severe FI).

A multinomial logistic regression analysis was performed to analyze the association between the outcome variables (food security, MFI, and MSFI) and the perceived food environment. The relative risk ratios (RRRs) and corresponding confidence intervals (95% CI; *p* < 0.05) were estimated, adjusting for sex, self-reported skin color, age, education level, household income, and place of residence. This adjustment was based on scientific evidence [23] and statistical significance in the bivariate analysis (Chi-square test). Before running the model, multicollinearity was tested between the model’s adjustment variables. To this end, the VIF (Variance Inflation Factor) test was performed, and the redundant predictor was removed, in this case, the variable presence of children under ten years of age in the household. All analyses were conducted using STATA 16.0 statistical software, considering the sample weights and model fit testing. 

The present study was approved by the Research Ethics Committee of the Clementino Fraga Filho University Hospital at UFRJ (Hospital Universitário Clementino Fraga Filho) (CAEE: 54473421.6.0000.5257), and it was conducted according to all ethical guidelines for human studies in the country.

## 3. Results

Table 1 presents the sociodemographic and economic characteristics according to the prevalence of FI among households. The final sample used in this study consisted of a total of 1819 households (with 181 interviews lost; 9.05%). The majority of household heads were female (53.3%), self-declared black (71.7%), and had completed high school (43.4%). Moderate/severe FI was significantly higher among households where the head of the household was self-declared black (84.7%), had a lower level of education (58.8%), and had an income below ½ minimum wage (56.5%).

Most household heads perceived that, in their neighborhoods, fruits and vegetables were easily accessible (95.0%), of good quality (94.1%), and of a wide variety (91.5%). Regarding ultra-processed foods, most reported that these products were widely available (94.0%) and of a large variety (94.8%). In terms of food prices, 70.0% perceived ultra-processed foods as affordable in their neighborhoods, while less than half (49.0%) of household heads considered fruits to be affordably priced in their region. Still related to food prices, fast food meals were deemed cheap by 62.3% of household heads. When analyzing perceptions according to FI levels, a clear gradient was observed: as FI levels increase, the perception of the food environment worsens. Families experiencing MSFI had a poorer perception of the ease of access (86%), variety (85.5%), quality (83.6%), and affordability (31.7%) of FVs (Table 2).

**Figure 1:**
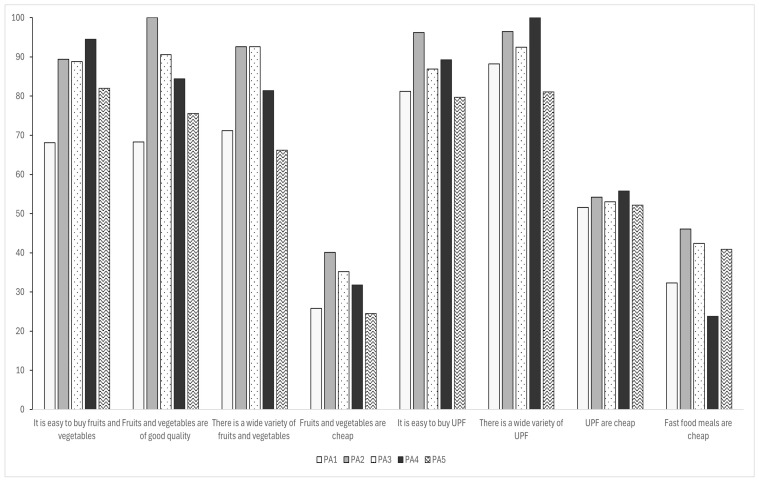
Perceptions of the food environment among families experiencing Moderate or Severe Food Insecurity (MSFI) across planning areas. Rio de Janeiro, 2024.

When analyzing the relationship between the perception of the food environment and FI levels and considering place of residence (using PAs as the geographic measure), differences in food environment perceptions were observed (Figure 1). Noticeably, household heads in PA1 had the poorest perception of their neighborhood in terms of availability (68.1%), quality (68.3%), and affordability of FV (25.8%). Conversely, residents of PA4 had the most positive assessment of food availability, with 94.5% of residence chiefs reporting easy access to FVs. Regarding food quality, PA3 household heads were the ones who best evaluated their area (90.6%). For UPFs, more than 50% of families across all PAs considered them (Table S1).

Table 3 presents the results of the association between food environment perception and FI levels. The adjusted multinomial regression revealed a negative perception regarding FV availability, quality, and affordability, which remained associated with mild FI and moderate/severe FI, where households that disagreed that it was easy to buy FV had a 5.6 times higher chance of experiencing moderate/severe FI (MSFI OR = 5.6 [95% CI: = 3.0 10.4).

## 4. Discussion

The results highlighted that a negative perception of FV availability, affordability, and quality had a direct association with FI. This fact corroborates the hypothesis that neighborhoods with fewer retail points for fresh and minimally processed foods reduce the dimensions of availability and accessibility of food security. As a result, these neighborhoods tend to have greater chances of households experiencing different levels of FI.

The FI indicator, particularly in its moderate and severe forms, serves as a measure of social inequity, highlighting the many inequalities present in the city. In households across the municipality of Rio de Janeiro (MRJ), 32.9% experienced some level of FI, with 16% facing moderate and severe FI, affecting nearly one million residents (990,849 people). The profile associated with FI in MRJ aligns with findings from national and state-level studies [25,27,38]. Access to adequate and quality food is marked by inequality, in which women, black people, those with low education levels, informal workers, and low-income families present worse FI conditions. All these results show that the dynamics of income distribution are a fundamental conditioner of FI dynamics, pointing to a strong relationship between FI and poverty, especially in metropolitan regions.

A territory with a wide availability of retail points for fresh and minimally processed foods aligns with two key dimensions of food security: (a) availability, which assesses whether food is physically present in the area, and (b) access, which relates to the physical and economic accessibility of these kinds of foods for families and individuals [39]. This study analyzed both dimensions and found that, although FVs are generally available in neighborhoods across Rio de Janeiro, they are not economically accessible. This highlights the high cost of fresh and minimally processed foods as a potential barrier to food security in these areas. Moreover, the analysis of associations revealed that a negative perception of the food environment—regarding FV availability, affordability, and variety—increased the likelihood of households experiencing FI.

It is important to contextualize that Rio de Janeiro has positioned itself as one of the most expensive Brazilian capitals for food consumption. Between December 2023 and February 2024, the period during which the data collection for this study took place, the cost of the basic food basket in the municipality of Rio de Janeiro increased by 12.8%, making it the most expensive in the country, according to data monitored by the Inter-Union Department of Statistics and Socio-Economic Studies (DIEESE) [40]. When analyzing the National Index of Price to the Ample Consumer (IPCA), an indicator used by the Brazilian Institute of Geography and Statistics (IBGE) to measure inflation, for January 2024, Rio de Janeiro ranked as the fifth highest capital with the highest IPCA increase, surpassing the national average [41]. The high cost of food has significantly increased the cost of living, pushing a portion of citizens into a state of vulnerability and making them highly susceptible to moderate/severe FI.

Considering the FI levels within the population, notable differences were observed in perceptions of the food environment across all analyzed dimensions. As the FI of the families worsened, so did the perception of the food environment. Household heads experiencing moderate/severe FI perceived lower availability, higher prices, and poorer quality of FVs in their neighborhoods compared to those living in food security. This scenario may be associated with a double load of vulnerability, where families already struggling to regularly access sufficient food in quality and quantity, are also facing territorial barriers that impose difficulties in accessing food—whether in terms of availability, quality, or affordability. Studies in different parts of the world, including countries with better socioeconomic conditions than Brazil, also indicate this double vulnerability in access to food and territory [42,43].

When analyzing perceptions of the food environment in terms of the availability and economic accessibility of UPFs, we identified that they are just as available as FVs in neighborhoods. However, they are perceived as cheaper, even by household heads experiencing FI. This high availability and low cost of UPFs encourage their consumption [44,45], which, in turn, is linked to negative health outcomes, such as obesity and other chronic non-communicable diseases (NCDs) [46,47,48]. Notably, there is an increasing amount of scientific evidence pointing to an association of FI with obesity and NCDs [18,49], both in Brazil and in several other countries that present high patterns of social inequalities [50,51].

Research increasingly suggests that healthier food choices tend to be more expensive than unhealthy ones [52,53]. Additionally, studies indicate the growing availability of food via delivery services, where UPFs tend to have greater discounts compared to FVs [54,55,56]. These findings align with our results, showing a higher agreement that fast food meals are affordable in neighborhoods compared to FVs.

Low income is also a key determinant of FI [57,58], as food prices play a crucial role in determining food acquisition, especially for income-deprived families [59,60,61]. In this context, families in Rio de Janeiro who perceive their neighborhoods as more favorable to UPFs than to fresh and minimally processed foods—while also experiencing FI—experience a context favorable for multiple forms of malnutrition.

One of the consequences of this scenario is the possible worsening of inequalities in major metropolitan areas in Latin America, as is the case of Rio de Janeiro, particularly in low- and middle-income countries,. In this regard, socio-spatial segregation has been identified as a fundamental driver of disparities in access to healthy food options [62,63,64]. Some authors emphasize that the high availability and low cost of UPFs deepen social, racial, and territorial inequalities in food and nutrition outcomes [62,65]. These factors facilitate the consumption of UPFs among the most vulnerable populations, particularly low-income and black communities [44]. This reality—where cheaper, unhealthy foods are more accessible for more vulnerable socioeconomically groups concentrated in specific urban areas—is associated with a higher prevalence of outcomes such as FI, obesity, and NCDs in these same populations [44,66], and has led some researchers to coin the concept of food apartheid [67,68].

It is noteworthy that the programmatic areas of Rio de Janeiro where household heads perceived a worse food environment also have the highest prevalence of moderate/severe FI [22], lower availability of healthcare services [69], greater racial and economic segregation [70], and higher concentrations of food deserts around schools [71]. These findings indicate a structural socio-spatial segregation within the city of Rio, which isolates part of the population from essential goods and services—ultimately undermining their fundamental human right to adequate and healthy food. This structural socio-spatial segregation is important when considering how segregated Latin American cities are, especially the large urban metropolises of the continent. The segregated makeup of these cities, including Rio de Janeiro, would benefit from a strategy of territorial integration of the provision of access to services and food, further highlighting the social inequalities arising from spatial segregation in these cities [72].

The association found between perceptions of the food environment and moderate/severe FI in the city of Rio de Janeiro points to important challenges in the implementation of public policies in the municipality. Addressing FI involves improving access to healthy foods, and the results presented here indicate that residents of more vulnerable territories who perceive their food environment as unfavorable are more susceptible to moderate/severe insecurity. It is imperative that the municipal government consider strategies to facilitate access to healthy foods, especially through territorialized policies that help to reduce inequalities. In this sense, the results presented here may help public managers to focus on actions that are planned in the first municipal food security plan for the municipality, especially in the most critical planning areas, such as the implementation of health facilities in food desert areas, the increase in the implementation of income transfer programs, and the expansion of programs such as popular restaurants and community kitchens [73].

The comparison of our findings with the existing literature is challenging due to the heterogeneity of the measurement instruments used to assess perceptions of the food environment [7]. Only two studies were found that analyzed the association between the food environment and FI levels using the same exposure and outcome scales as this study and, similarly to our results, they identified a direct association between the perceived environment and FI levels. However, it is important to note that these studies sought to analyze the association between the food environment and FI levels focused on households with specific populations—schoolchildren in two medium-sized Brazilian cities. Within this context, a negative perception of food quality and price was found to be associated with moderate/severe FI [23,74].

By comparing the results of the present study with others that also assessed the perceived food environment, but used different instruments to measure neighborhood perceptions, it was found that, in general, the studies had similar results to ours. Associations were found between FI and the perception of access to more expensive FVs [75,76], low-quality FVs [77,78], and limited access to food [75,77,78,79].

Although the concept of the neighborhood constructed in the study sought to integrate geographic and sociodemographic characteristics, the measurement of this construct may have suffered from inaccuracies. The average values of neighborhood perceptions (analyzed exposures) were created based on the responses of individuals within the same geographic unit. However, perception may not correspond to the actual evaluated space, as individuals may have different notions of what constitutes a neighborhood [80]. Another criticism of studies using neighborhood perceptions is related to the self-selection bias, meaning that individuals choose their place of residence based on characteristics they consider important. However, in countries with significant social inequalities, such as Brazil, only a very small portion of the population has the ability to choose where they live [81,82]. Another limitation is the geographical representativeness of the study, which used a spatial unit larger than the neighborhood to construct the sample [83].

We would also like to highlight the strengths of the study, which followed a random and stratified sampling process representative of the population of the municipality of Rio de Janeiro and used the full version of the EBIA, an instrument validated for the Brazilian population. Additionally, this is the first study to propose evaluating the association between perceptions of the food environment and FI in a representative sample of a major Brazilian metropolis, addressing a scientific gap identified in recent reviews [84].

## 5. Conclusions

The contributions of this study include providing evidence on the association between perceptions of the food environment and levels of food security in households within a metropolis of a middle-income country, characterized by a context of significant inequality. The results show that families experiencing FI perceive that healthy foods are available in their area, but are expensive and offered in less variety. Additionally, they indicate that UPFs are more available and varied than FVs for the general population.

Thus, inequity in FI is comprises differences in access to food and healthy eating, conditions which are socially produced and have a negative impact on the well-being and quality of life of families and their members. Public policies that focus both on social protection and income security, as well as on strengthening and promoting healthier food environments, can influence healthy eating and help reduce disparities related to food and nutrition, especially in large metropolises.

Therefore, investment in intersectoral policies that strengthen the entire food production and distribution chain of fresh and minimally processed foods, while facilitating their availability at food sales points, especially in socially vulnerable areas, has proven to be an important approach to mitigating food-related issues. It is also crucial in containing the spread of UPFs in these areas, and to this end, the adoption of protective public policies such as taxation and the banning of advertising for these foods has been effective.

## Figures and Tables

**Table 1:** Distribution of sociodemographic characteristics according to the total sample (n = 1819) and the levels of household food security and insecurity (FI)—Rio de Janeiro, 2024.

	All	Food Security	Mild Food Insecurity (MFI)	Moderate or Severe Food Insecurity (MSFI)	*p*-Value *
	% (95%CI)	% (95%CI)	% (95%CI)	% (95%CI)	
**Head of household characteristics**					
**Sex**					
Male	46.7 (44.04, 49.30)	51.6 (48.43, 54.70)	35.7 (29.54, 42.30)	38.3 (31.84, 45.22)	**<0.01**
Female	53.3 (50.70, 55.96)	48.4 (45.30, 51.57)	64.3 (57.70, 70.46)	61.7 (54.78, 68.16)	
**Race/Skin color**					
White	28.3 (25.97, 30.69)	32.1 (29.26, 35.10)	25.6 (20.08, 32.12)	15.3 (11.16, 20.70)	**<0.01**
Black	71.7 (69.31, 74.03)	67.9 (64.90, 70.74)	74.4 (67.88, 79.92)	84.7 (79.30, 88.84)	
**Age**					
18–35	23.1 (20.92, 25.35)	22.0 (19.54, 24.70)	27.8 (22.25, 34.20)	22.3 (17.08, 28.61)	0.21
36–45	17.7 (15.84, 19.76)	18.5 (16.25, 20.94)	17.0 (12.69, 22.46)	15.3 (10.78, 21.20)	
46–60	25.6 (23.33, 27.93)	24.2 (21.63, 27.02)	26.5 (21.04, 32.69)	30.1 (24.20, 36.77)	
60 or older	33.7 (31.22, 36.21)	35.3 (32.34, 38.33)	28.7 (22.96, 35.19)	32.3 (26.12, 39.16)	
**Education level**					
Elementary	33.6 (31.13, 36.24)	25.3 (22.60, 28.15)	44.2 (37.62, 51.01)	58.8 (51.50, 65.83)	**<0.01**
High school	43.4 (40.79, 46.09)	44.9 (41.80, 48.07)	45.7 (39.07, 52.53)	34.2 (27.69, 41.45)	
University	22.9 (20.86, 25.16)	29.8 (27.09, 32.68)	10.1 (6.77, 14.75)	6.9 (3.91, 11.91)	
**Family income per capita (minimum wage)**					
Up to 1/2	21.6 (19.27, 24.04)	7.6 (5.92, 9.58)	38.4 (31.90, 45.29)	56.5 (49.40, 63.32)	**<0.01**
1/2 to 1	29.7 (27.17, 32.35)	27.2 (24.27, 30.41)	40.0 (33.44, 46.84)	28.2 (22.31, 34.95)	
More than 1 up to 2	27.3 (24.84, 29.79)	33.5 (30.36, 36.80)	17.4 (13.04, 22.76)	14.1 (9.92, 19.70)	
More than 2	21.5 (19.42, 23.74)	31.7 (28.75, 34.84)	4.3 (2.35, 7.76)	1.2 (0.34, 4.02)	

* Statistically significant associations based on Pearson’s Chi-square test (*p* < 0.05). 95%CI: 95% confidence interval.

**Table 2:** Dimensions of perceptions of the food environment in the neighborhood according to the total sample (n = 1819) and the level of household food security and insecurity (FI)—Rio de Janeiro, 2024.

Dimensions		Total	Food Security	Mild Food Insecurity (MFI)	Moderate or Severe Food Insecurity (MSFI)	*p*-Value *
		% (95%CI)	% (95%CI)	% (95%CI)	% (95%CI)	
It is easy to buy fruits and vegetables	Agree	95.0 (93.7–96.0)	97.9 (96.9–98.6)	92 (87.3–95.1)	86.0 (80.5–90.1)	<0.01
	Disagree	5.0 (3.9–6.2)	2.1 (1.4–3.1)	8.0 (4.9–12.7)	14.0 (9.9–19.6)	
Fruits and vegetables are of good quality	Agree	94.1 (92.7–95.3)	96.9 (95.6–97.8)	90.7 (85.7–94.0)	85.5 (79.8–89.8)	<0.01
	Disagree	5.9 (4.7–7.3)	3.1 (2.2–4.4)	9.3 (6.0–14.3)	14.5 (10.2–20.2)	
There is a wide variety of fruits and vegetables	Agree	91.5 (89.9–92.9)	94.2 (92.5–95.5)	88.2 (83.0–91.9)	83.6 (77.8–88.1)	<0.01
	Disagree	8.5 (7.1–10.1)	5.9 (4.5–7.5)	11.9 (8.1–17.1)	16.4 (11.9–22.1)	
Fruits and vegetables are cheap	Agree	48.9 (46.2–51.6)	54.4 (51.1–57.5)	44.3 (37.5–51.3)	31.7 (25.3–38.8)	<0.01
	Disagree	51.1 (48.4–53.8)	45.6 (42.5–48.9)	55.7 (48.7–62.5)	68.3 (61.2–74.7)	
It is easy to buy UPFs	Agree	94.0 (92.5–95.1)	95.7 (94.1–96.9)	94.7 (90.6–97.0)	85.5 (79.4–90.0)	<0.01
	Disagree	6.1 (4.8–7.5)	4.3 (3.1–5.8)	5.3 (3.0–9.4)	14.5 (10.0–20.6)	
There is a wide variety of UPFs	Agree	94.8 (93.4–95.9)	95.7 (94.1–96.9)	95.6 (91.7–97.7)	89.9 (84.6–93.5)	<0.01
	Disagree	5.2 (4.1–6.7)	4.3 (3.1–5.9)	4.4 (2.3–8.3)	10.2 (6.5–15.4)	
UPFs are cheap	Agree	69.4 (66.7–71.9)	72.2 (69.2–75.0)	74.1 (67.4–79.9)	52.9 (45.4–60.3)	<0.01
	Disagree	30.6 (28.1–33.3)	27.8 (25.0–30.8)	25.9 (20.1–32.6)	47.1 (39.7–54.6)	
Fast food meals are cheap	Agree	62.3 (59.4–65.0)	69.8 (66.6–72.8)	55.2 (47.8–62.3)	40.8 (33.5–48.5)	<0.01
	Disagree	37.8 (35.0–40.6)	30.2 (27.2–33.4)	44.8 (37.7–52.2)	59.2 (51.5–66.5)	

Notes: * Statistically significant associations based on Pearson’s Chi–square test (*p* < 0.05). UPFs: ultra-processed foods, 95%CI: 95% confidence interval.

**Table 3:** Adjusted relative risk ratios (RRRs) and confidence intervals between the dimensions of perceptions of the food environment in the neighborhood and household food security and food insecurity (FI) levels in the municipality of Rio de Janeiro, 2024.

Dimensions		Perception of the Food Environment			
		Mild FI (MFI)		Moderate or Severe FI (MSFI)	
		Raw	Adjusted **	Raw	Adjusted **
		RRR (95%CI)	RRR (95%CI)	RRR (95%CI)	RRR (95%CI)
It is easy to buy fruits and vegetables	Agree	1.0	1.0	1.0	1.0
	Disagree	3.1 (1.8–5.3)	**2.2 (1.2–4.2)**	7.6 (4.8–12.0)	**5.6 (3.0–10.4)**
Fruits and vegetables are of good quality	Agree	1.0	1.0	1.0	1.0
	Disagree	2.7 (1.6–4.4)	**2.0 (1.1–3.5)**	5.9 (3.8–9.2)	**4.5 (2.6–7.9)**
Fruits and vegetables are cheap	Agree	1.0	1.0	1.0	1.0
	Disagree	1.2 (0.9–1.6)	**1.5 (1.1–2.0)**	2.2 (1.6–3.0)	**2.5 (1.7–3.6)**
There is a wide variety of fruits and vegetables	Agree	1.0	1.0	1.0	1.0
	Disagree	2.0 (1.3–3.1)	1.5 (0.5–1.3)	3.8 (2.6–5.6)	**2.7 (1.6–4.4)**
It is easy to buy UPFs	Agree	1.0	1.0	1.0	1.0
	Disagree	1.6 (0.9–2.9)	1.2 (0.6–2.3)	4.4 (2.8–7.0)	**2.6 (1.4–4.7)**
UPFs are cheap	Agree	1.0	1.0	1.0	1.0
	Disagree	0.9 (0.6–1.2)	1.0 (0.7–1.4)	2.1 (1.5–2.7)	**2.0 (1.3–2.8)**
There is a wide variety of UPFs	Agree	1.0	1.0	1.0	1.0
	Disagree	1.3 (0.7–2.4)	0.8 (0.4–1.7)	2.8 (1.7–4.6)	1.5 (0.8–2.8)
Fast food meals are cheap	Agree	1.0	1.0	1.0	1.0
	Disagree	1.5 (1.2–2.1)	**1.6 (1.2–2.3)**	3.2 (2.4–4.3)	**3.1 (2.1–4.5)**

Notes: minimum wage: in the year of research, minimum wage in Brazil was R$1320.00 (equivalent to approximately USD 263.96). CI: confidence interval; MFI: Mild Food Insecurity; and MSFI: Moderate or Severe Food Insecurity. The raw data were adjusted with the following variables: age, household income per capita, education level, planning area, and having a child under 10 years old. ** The Akaike Information Criterion (AIC) for adjusted model was 2156, with CI95%.

## Data Availability

The original contributions presented in this study are included in the article/Supplementary Materials. Further inquiries can be directed to the corresponding author.

## Supplementary Materials

The following supporting information can be downloaded at: https://www.mdpi.com/article/10.3390/ijerph22040642/s1, Table S1: Perception of the food environment according to the five planning areas and the level of food insecurity among households
