# Supplementary material for: Perception of the Food Environment and Food Security Levels of Residents of the City of Rio de Janeiro
Source: Int J Environ Res Public Health. 2025 Apr 18;22(4):642. doi: 10.3390/ijerph22040642 (PMC12026548; doi:10.3390/ijerph22040642)
Supplement: Supplementary file 1 [file ijerph-22-00642-s001.zip › ijerph-3558057-supplementary.pdf]

Supplementary Table. Perception of the food environment according to the five planning areas and the level of food insecurity among households.

Variables		PA1 %FS (95%CI)	PA1 %MFI (95%CI)	PA1 %MSFI (95%CI)	PA1 p-value*	PA2 %FS (95%CI)	PA2 %MFI (95%CI)	PA2 %MSFI (95%CI)	PA2 p-value*
It is easy to buy fruits and vegetables	Agree	93.2 (89.0;95.9)	86.3 (76.3;92.5)	68.1 (56.2;78.0)	< 0.01	98.3 (96.0;99.3)	100,0	89.4 (71.6;96.6)	< 0.01
	Disagree	6.8 (4.1;11.0)	13.7 (7.5;23.7)	31.9 (22.0;43.8)		1.7 (0.7;4.0)	0,0	10.7 (3.4;28.4)	
Fruits and vegetables are of good quality	Agree	92.7 (88.4;95.5)	88.4 (78.4;94.1)	68.3 (55.8;78.6)	< 0.01	99.0 (96.9;99.7)	95.3 (83.1;98.8)	100,0	0.12
	Disagree	7.3 (4.5;11.6)	11.6 (5.9;21.6)	31.8 (21.4;44.2)		1.0 (0.3;3.1)	4.7 (12.0;17.0)	0,0	
There is a wide variety of fruits and vegetables	Agree	90.4 (85.6;93.6)	87.3 (77.3;93.3)	71.2 (59.2;80.9)	< 0.01	97.6 (95.1;98.9)	95.1 (82.3;98.8)	92.6 (74.8;98.2)	0.26
	Disagree	9.6 (6.3;14.3)	12.7 (6.7;22.7)	28.8 (19.1;40.9)		2.4 (1.1;4.9)	4.9 (1.2;17.7)	7.4 (1.8;25.2)	
Fruits and vegetables are cheap	Agree	44.4 (37.7;51.3)	47.1 (35.5;58.9)	25.8 (16.6;37.7)	0.02	34.9 (29.5;40.7)	51.1 (35.8;66.3)	40.1 (23.0;59.9)	0.14
	Disagree	55.6 (48.7;62.3)	52.9 (41.1;64.5)	74.2 (62.3;83.4)		65.1 (59.3;70.5)	48.9 (33.7;64.3)	60.0 (40.1;77.0)	
It is easy to buy UPF	Agree	98.2 (95.2;99.3)	91.7 (82.6;96.2)	81.2 (70.1;88.8)	< 0.01	96.9 (94.2;98.4)	100,0	96.2 (77.2;99.5)	0.50
	Disagree	1.8 (0.7;4.8)	8.3 (3.8;17.4)	18.8 (11.2;29.9)		3.1 (1.6;5.8)	0,0	3.8 (0.5;22.8)	
There is a wide variety of UPF	Agree	97.2 (94.0;98.8)	91.4 (82.6;96.1)	88.2 (78.1;94.0)	< 0.01	96.3 (93.4;97.9)	100,0	96.5 (78.5;99.5)	0.44
	Disagree	2.8 (1.2;6.0)	8.6 (3.9;17.9)	11.8 (6.0;21.9)		3.7 (2.1;6.6)	0,0	3.6 (0.5;21.5)	
UPF are cheap	Agree	75.0 (68.4;80.6)	73.5 (61.8;82.7)	51.6 (39.4;63.5)	< 0.01	60.2 (54.1;65.9)	76.5 (59.5;87.8)	54.2 (34.6;72.6)	0.14
	Disagree	25.0 (19.4;31.6)	26.5 (17.3;38.2)	48.4 (36.5;60.6)		39.8 (34.1;45.9)	23.5 (12.2;40.5)	45.8 (27.4;65.5)	
Fast food meals are cheap.	Agree	63.9 (56.7;70.6)	60.3 (47.8;71.6)	32.3 (21.8;44.9)	< 0.01	55.4 (49.1;61.6)	56.6 (40.4;71.5)	46.1 (28.3;65.0)	0.65
	Disagree	36.1 (29.4;43.3)	39.7 (28.4;52.2)	67.7 (55.1;78.2)		44.6 (38.4;51.0)	43.4 (28.5;59.6)	53.9 (35.0;71.7)	

(continues)

Supplementary Table (continued)

Variables		PA3 %FS (95%CI)	PA3 %MFI (95%CI)	PA3 %MSFI (95%CI)	PA3 p-value*	PA4 %FS (95%CI)	PA4 %MFI (95%CI)	PA4 %MSFI (95%CI)	PA4 p-value*
It is easy to buy fruits and vegetables	Agree	99.0 (96.0;99.8)	88.5 (79.9;93.7)	88.8 (79.8;94.1)	< 0.01	97.5 (95.0;98.7)	94.5 (80.4;98.6)	94.5 (69.4;99.2)	0.50
	Disagree	1.0 (0.3;4.0)	11.5 (6.3;20.1)	11.2 (5.9;20.2)		2.6 (1.3;5.0)	5.5 (1.4;19.7)	5.5 (0.8;30.6)	
Fruits and vegetables are of good quality	Agree	96.9 (93.2;98.6)	88.1 (79.3;93.5)	90.6 (81.5;95.5)	0.01	95.5 (92.5;97.3)	91.5 (76.6;97.2)	84.4 (61.0;94.9)	0.08
	Disagree	3.1 (1.4;6.8)	11.9 (6.5;20.7)	9.4 (4.5;18.5)		4.5 (2.7;7.5)	8.5 (2.8;23.4)	15.6 (5.1;39.0)	
There is a wide variety of fruits and vegetables	Agree	93.8 (89.4;96.5)	87.3 (78.4;92.8)	92.6 (84.4;96.6)	0.17	92.5 (88.9;95.0)	84.8 (68.2;93.6)	81.4 (55.4;93.9)	0.12
	Disagree	6.2 (3.5;10.6)	12.7 (7.2;21.6)	6.3 (3.4;15.6)		7.5 (5.0;11.1)	15.2 (6.44.31.8)	18.6 (6.1;44.6)	
Fruits and vegetables are cheap	Agree	67.5 (60.4;73.8)	44.2 (33.9;55.0)	35.2 (25.5;46.3)	< 0.01	41.2 (35.6;47.0)	20.1 (9.9;36.7)	31.8 (15.0;55.2)	0.05
	Disagree	32.6 (26.3;39.6)	55.9 (45.0;66.2)	64.8 (53.7;74.5)		58.8 (53.0;64.4)	79.9 (63.4;90.1)	68.2 (44.8;85.0)	
It is easy to buy UPF	Agree	94.8 (90.6;97.2)	93.9 (86.1;97.5)	86.9 (77.3;92.8)	0.07	95.4 (92.3;97.3)	86.1 (70.6;94.1)	89.3 (65.8;97.3)	0.06
	Disagree	5.2 (2.8;9.4)	6.1 (2.6;13.9)	13.1 (7.2;22.7)		4.6 (2.8;7.7)	13.9 (5.9;29.5)	10.7 (2.7;34.2)	
There is a wide variety of UPF	Agree	94.3 (90.0;96.8)	95.2 (87.9;98.2)	92.5 (84.2;96.6)	0.75	96.0 (93.1;97.7)	94.2 (79.3;98.5)	100,0	0.56
	Disagree	5.7 (3.2;10.1)	4.8 (1.8;12.1)	7.5 (3.4;15.9)		4.0 (2.3;6.9)	5.9 (1.5;20.7)	0,0	
UPF are cheap	Agree	79.8 (73.3;85.0)	73.8 (63.3;82.1)	53.0 (42.0;6.8)	< 0.01	61.7 (55.8;67.3)	53.5 (35.9;70.3)	55.8 (33.1;76.2)	0.62
	Disagree	20.2 (15.0;26.7)	26.3 (17.9;36.7)	47.0 (36.2;58.0)		38.3 (32.8;44.2)	46.5 (29.7;64.1)	44.3 (23.8;66.9)	
Fast food meals are cheap.	Agree	77.5 (70.2;83.4)	53.4 (42.4;64.1)	42.4 (31.5;54.0)	< 0.01	63.2 (57.1;69.0)	44.4 (27.1;63.2)	23.8 (9.2;49.1)	<0.01
	Disagree	22.5 (16.6;29.8)	46.6 (36.0;57.6)	57.7 (46.1;68.5)		36.8 (31.1;42.9)	55.6 (36.8;72.9)	76.2 (50.9;90.8)	

(continues)

Supplementary Table (continued)

Variables		PA5			
		%FS (95%CI)	%MFI (95%CI)	%MSFI (95%CI)	p-value*
It is easy to buy fruits and vegetables	Agree	97.5 (94.5;98.9)	96.3 (86.4;99.1)	82.0 (70.3;89.8)	< 0.01
	Disagree	2.5 (1.1;5.5)	3.7 (0.9;13.7)	18.0 (10.2;29.7)	
Fruits and vegetables are of good quality	Agree	97.0 (93.9;98.6)	94.4 (83.8;98.2)	75.6 (62.8;85.1)	<0.01
	Disagree	3.0 (1.4;6.1)	5.6 (1.8;16.2)	24.4 (14.9;37.2)	
There is a wide variety of fruits and vegetables	Agree	93.7 (89.8;96.2)	88.3 (76.2;94.7)	66.2 (52.8;77.4)	<0.01
	Disagree	6.3 (3.8;10.2)	11.7 (5.3;23.8)	33.8 (22.6;47.2)	
Fruits and vegetables are cheap	Agree	63.5 (57.0;69.6)	50.0 (36.0;64.0)	24.5 (15.1;37.2)	<0.01
	Disagree	36.5 (30.4;43.0)	50.0 (36.0;64.0)	75.5 (62.8;85.0)	
It is easy to buy UPF	Agree	95.7 (92.1;97.7)	98.0 (86.8;99.7)	79.7 (66.1;88.8)	<0.01
	Disagree	4.4 (2.4;7.9)	2.0 (0.3;13.2)	20.3 (11.3;33.9)	
There is a wide variety of UPF	Agree	96.5 (93.1;98.3)	95.9 (84.8;99.0)	81.1 (68.2;89.6)	<0.01
	Disagree	3.5 (1.6;6.9)	4.1 (1.0;15.2)	18.9 (10.4;31.8)	
UPF are cheap	Agree	79.4 (73.1;84.6)	82.5 (67.5;91.5)	52.2 (38.1;65.9)	< 0.01
	Disagree	20.6 (15.5;26.9)	17.5 (8.5;32.5)	47.8 (34.1;61.9)	
Fast food meals are cheap.	Agree	77.4 (70.9;82.8)	61.9 (45.9;75.7)	40.9 (27.7;55.4)	< 0.01
	Disagree	22.6 (17.2;29.2)	38.1 (24.3;54.1)	59.1 (44.6;72.3)	

Note: 95% CI: 95% confidence interval
